# A case of acute kidney injury due to native kidney BK polyomavirus-associated nephropathy in a human T-lymphotropic virus type 1 carrier

**DOI:** 10.1186/s12882-023-03373-1

**Published:** 2023-10-31

**Authors:** Keita Takae, Yuki Ueno, Masumi Shojima, Hiroshi Nagae, Takako Nakano, Shohei Takata, Ritsuko Katafuchi, Kosuke Masutani, Toshiaki Nakano, Yusuke Kuroki

**Affiliations:** 1 Division of Nephrology, National Hospital Organization Fukuokahigashi Medical Center, 1-1-1 Chidori, Koga City, 811-3195 Japan; 2 Division of Respiratory Medicine, National Hospital Organization Fukuokahigashi Medical Center, Koga, Japan; 3 Kano Hospital, Shingu, Japan; 4 Division of Nephrology and Rheumatology, Department of Internal Medicine, Faculty of Medicine, Fukuoka University, Fukuoka, Japan; 5 Department of Medicine and Clinical Science, Graduate School of Medical Sciences, Kyushu University, Fukuoka, Japan

**Keywords:** BK polyomavirus, Kidney injury, Human T-lymphotropic virus type 1

## Abstract

**Background:**

BK polyomavirus-associated nephropathy (BKPyVAN) has become a major cause of kidney dysfunction and graft loss in kidney transplant recipients. On rare occasion, polyomavirus has also been known to affect native kidneys of immunocompromised individuals. Only a small number of opportunistic infections have been reported in the carrier phase of human T-lymphotropic virus type 1 (HTLV-1). This is the first reported case of BKPyVAN in native kidneys of an HTLV-1 carrier.

**Case presentation:**

A 61-year-old man was referred to our hospital from a primary care physician for work-up and treatment of pneumonia. He was diagnosed with *Pneumocystis* pneumonia and identified as a HTLV-1 carrier who had not yet developed adult T-cell leukemia (ATL). The pneumonia was successfully treated with sulfamethoxazole-trimethoprim. He had never been diagnosed with any kind of kidney dysfunction. Laboratory investigations showed a serum creatinine of 5.3 mg/dL, and urinary sediment showed cells with nuclear enlargement and inclusion bodies suggesting viral infection. The urinary Papanicolaou stain showed inclusions in swollen, ground-glass nuclei, typical of “decoy cells”. Renal biopsy showed degeneration of tubules with epithelial enlargement, vacuolar degeneration, nuclear inclusion bodies, and detachment from the tubular basement membrane. Tubular nuclei showed positive staining positive for simian virus 40 large-T antigen. Polymerase chain reaction tests for BK polyomavirus DNA of both urine and plasma were positive. These findings confirmed a diagnosis of BKPyVAN. Intravenous immunoglobulin therapy did not improve renal function, necessitating maintenance hemodialysis therapy.

**Conclusions:**

BKPyVAN should be considered when acute kidney injury occurs with opportunistic infection. HTLV-1 carriers can develop opportunistic infections even before the onset of ATL.

## Background

BK polyomavirus (BKPyV) is a circular, double-stranded DNA virus from the polyomavirus family [1]. Primary BKPyV is typically acquired in childhood, with seroprevalence rates in adults reaching 80% [2]. In humans, primary infection typically occurs in childhood, as a respiratory viral infection. After primary infection, the virus establishes latency in the renal urinary epithelium and usually remains asymptomatic in immunocompetent hosts. The virus becomes reactivated in immunodeficiency states, typically in the allografts of kidney transplant recipients, with 30–58% of such patients who develop BKPyV-associated nephropathy (BKPyVAN) proceeding to graft failure [3]. As well as BKPyVAN, the BKPyV can cause ureteral stenosis in kidney transplant recipients, late-onset hemorrhagic cystitis and even bladder cancer in patients with allogeneic stem cell transplantation [1].

Human T-lymphotropic virus type 1 (HTLV-1), discovered in 1981 by Hinuma et al. [4], is a retrovirus that has been defined as a carcinogenic agent in humans. There are an estimated 10–20 million individuals with HTLV-1 infection worldwide, concentrated in endemic areas such as southwestern Japan, the Caribbean Islands, Africa, and South America [5]. In 1984, Yoshida et al. found that HTLV-1 caused adult T cell leukemia (ATL) [6]. The risks of asymptomatic HTLV-1 carriers developing ATL have been reported as 4–6% in men and 2.6% in women [7]. Patients with ATL are known as immunocompromised hosts. Recently, some cases of opportunistic infections such as *Pneumocystis* pneumonia or cryptococcosis in asymptomatic HTLV-1 carriers prior to onset of ATL have been reported [8].

We describe an otherwise healthy HTLV-1 carrier case who developed acute kidney injury due to native kidney BKPyVAN with *Pneumocystis* pneumonia. We believe this first report of such a case illustrates several important features of this disease.

## Case presentation

**Fig. 1:**
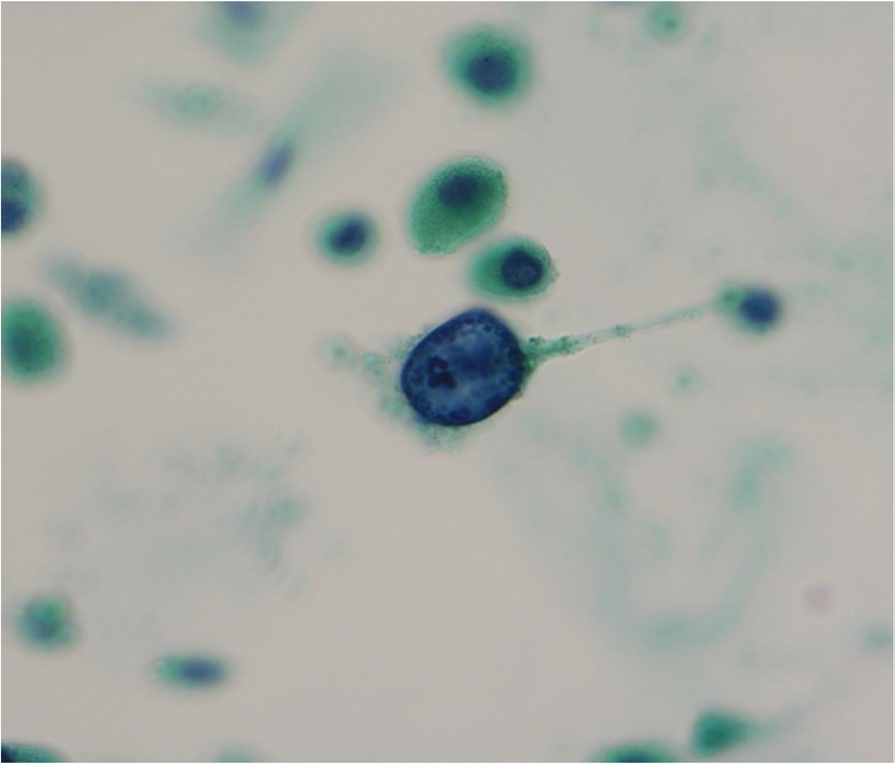
Urinary Papanicolaou stain showing decoy cells. Cells are characterized by inclusion bodies in swollen, ground-glass nuclei

**Fig. 2:**
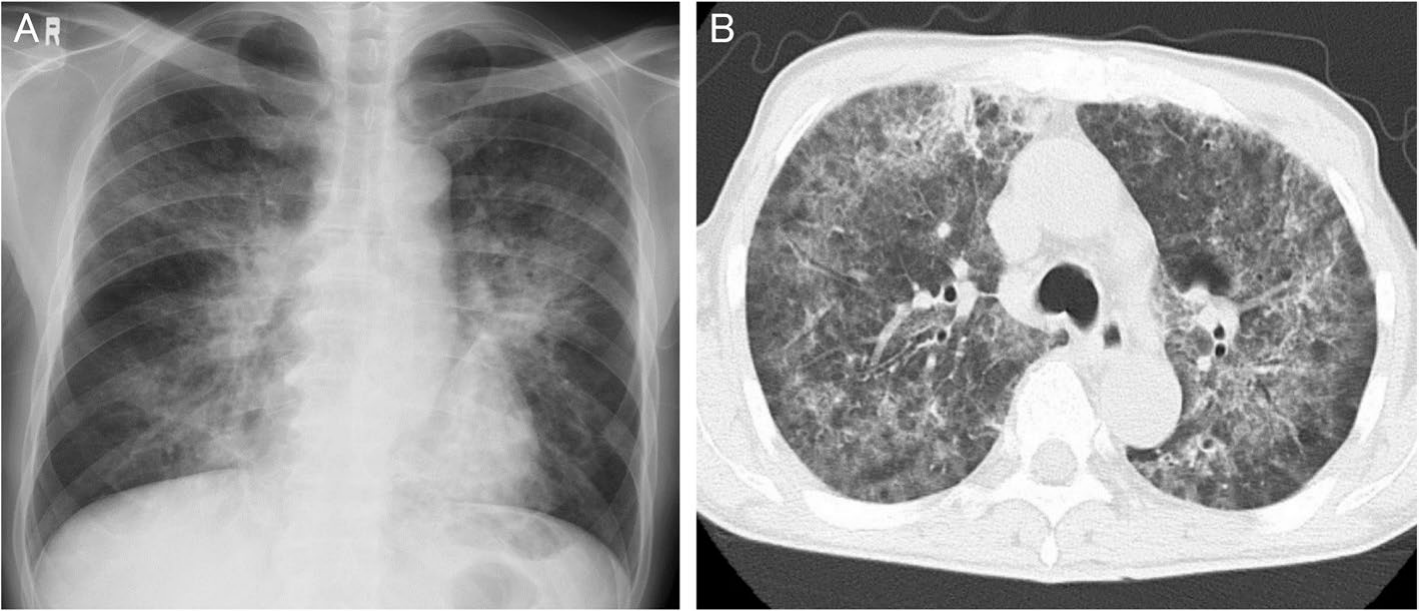
Chest radiographs. (**A**) Chest X-ray revealed infiltration shadows in bilateral middle and lower lung fields. (**B**) Chest computed tomography scan revealed infiltration and fine granular shadows

The patient was a 61-year-old Japanese man. He had been aware of gradually worsening dyspnea for one month. He visited a local clinic and was diagnosed with pneumonia, with referral to our hospital for treatment. He was admitted to our hospital on the day of referral. His initial vital signs were as follows: blood pressure, 116/77 mmHg; heart rate, 102 beats per minute; respiratory rate, 20 breaths per minute; SpO_2_, 98% in room air; and temperature, 36.7 °C. His white blood cell count was 12,800/mm^3^; neutrophil count, 9,900/mm^3^ (77.3%); lymphocyte count, 2,300/mm^3^ (18.1%); hemoglobin, 10.5 g/dL; platelet count, 400,000/mm^3^; total serum protein, 5.8 g/dL; albumin, 2.2 g/dL. No liver dysfunction was observed. His blood urea nitrogen (BUN) was 68 mg/dL; creatinine (Cr), 5.3 mg/dL; estimated glomerular filtration rate (eGFR), 9.6 mL/min/1.73m^2^; uric acid, 9.0 mg/dL; sodium, 135 mEq/L; potassium, 4.3 mEq/L; chloride, 101 mEq/L; calcium, 8.6 mg/dL; phosphate, 5.9 mg/dL; and c-reactive protein (CRP), 8.7 mg/dL. His urinalysis revealed granular casts but no red blood cells. The protein-to-creatinine ratio in his spot urine sample was 0.18 g/gCr. Cells in his urinary sediment showed nuclear enlargement and inclusion bodies, suggesting viral infection. The urinary Papanicolaou stain test revealed intranuclear inclusions in swollen, ground-glass nuclei, suggestive of decoy cells (Fig. 1). Additional laboratory results were as follows: rheumatoid factor, < 5 U/mL; IgG, 562 mg/dL; IgA, 60 mg/dL; IgM, 47 mg/dL; C3, 164 mg/dL; C4, 64 mg/dL; and CH50, 55 U/mL; he was negative for antinuclear antibody, anti-myeloperoxidase antibody, anti-proteinase 3 antibody, and anti-glomerular basement membrane antibody. Chest radiography showed an infiltration shadow in bilateral middle and lower lung fields (Fig. 2). His chest computed tomography scan revealed infiltration and fine granular shadows with air bronchogram (Fig. 2). Kidney ultrasonography showed no kidney atrophy or cortical thinning. Regarding pneumonia, β-D-glucan was high (300 pg/mL) and *Pneumocystis jirovecii* DNA was positive in bronchoalveolar lavage fluid. He was diagnosed with *Pneumocystis* pneumonia. Submitted sputum culture was positive for *α-hemolytic Streptococcus* and *Neisseria* spp. but were not determined to be the causative organism. No evidence of mycobacterium tuberculosis, atypical mycobacterial, bacterial, or SARS-CoV-2 viral pneumonia was found, and serological test was negative for anti-*Trichosporon asahii* antibody. Because the bronchoalveolar lavage test diagnosed *Pneumocystis* pneumonia, no other viral serological tests such as cytomegalovirus, measles, or varicella virus, which could cause pneumonia, were performed. An additional test was performed to scrutinize the cause of immunodeficiency. Human immunodeficiency virus (HIV) antibody was negative. The results revealed that the patient was infected with HTLV-1, with a high titer (≥ 256 times) of anti HTLV-1 antibodies and positive polymerase chain reaction for the HTLV-1 provirus. However, no atypical lymphocytes were observed in the peripheral blood and no notable findings were found in the bone marrow pathological findings. Additional pathological examination was not performed because no lymph node or skin lesion involvement was noted. Therefore, this patient was determined not to have developed ATL.

**Fig. 3:**
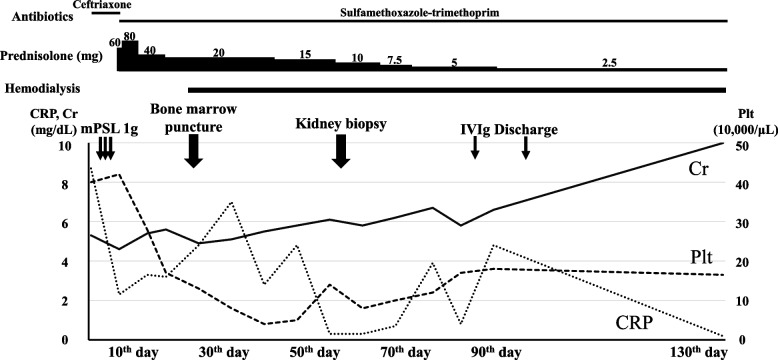
Clinical course. CRP, c-reactive protein; Cr, creatinine; IVIg, Intravenous immunoglobulin; mPSL, methylprednisolone; Plt, platelets

**Fig. 4:**
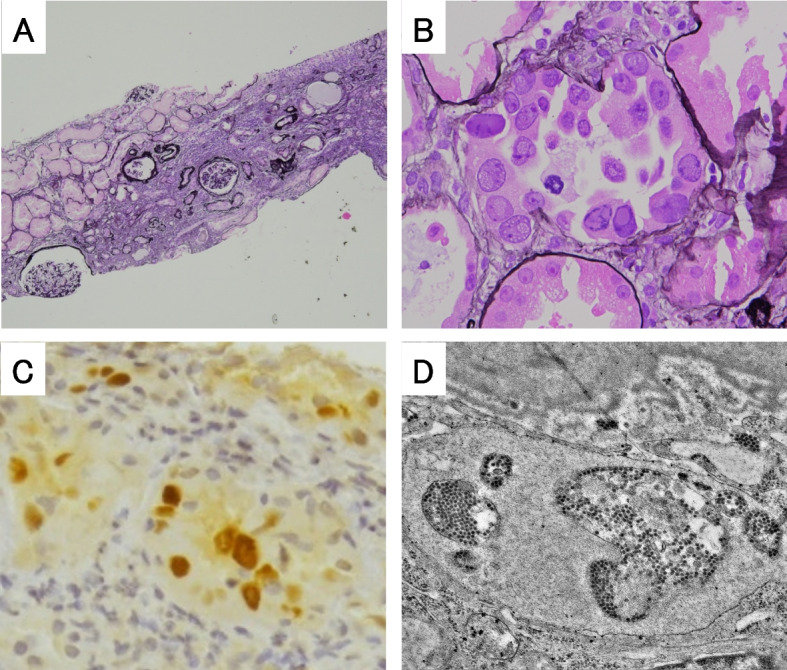
Histology of kidney biopsy specimen. (**A**) Tubular interstitium showed inflammation with lymphocytes and fibrosis. Tubular atrophy was seen diffusely (periodic acid methenamine silver stain, × 40). (**B**) Tubules showed enlargement, vacuolar degeneration, and detachment from the basement membrane (periodic acid methenamine silver stain, × 400). (**C**) SV-40-positive nuclei were found in tubular epithelial cells (anti-SV40 large-T antigen stain, × 400). (**D**) Intracellular spherical viral particles were seen in tubular epithelial cells (electron microscopy, × 8000)

His clinical course is shown in Fig. 3. Ceftriaxone was administered for pneumonia and was changed to trimethoprim-sulfamethoxazole after diagnosis of *Pneumocystis* pneumonia. Methylprednisolone 1 g per day was administered intravenously for 3 days and was switched to oral prednisolone afterwards. The patient did not require oxygen therapy on admission; however, respiratory distress worsened, and oxygen therapy was started on the third day of the hospitalization. Oxygen therapy was continued until hospital day 52. On hospital day 17, kidney function showed worsening (Cr, 5.6 mg/dL; eGFR, 9.1 mL/min/1.73m^2^) with an elevated BUN (122 mg/dL); therefore, hemodialysis therapy was initiated as an adjunct for infection control on hospital day 20. Kidney biopsy to determine the cause of acute kidney failure was considered but could not be performed immediately because of worsening inflammatory responses and drug-induced thrombocytopenia. Kidney biopsy performed on hospital day 54, after inflammatory response easing and platelet count recovery, showed diffuse degeneration of tubules with epithelial enlargement, vacuolar degeneration, nuclear inclusion bodies, and detachment from the tubular basement membrane (Fig. 4). Immunostaining for simian virus (SV) 40 large T-antigen was positive in tubular epithelial cell nuclei (Fig. 4). Electron microscopy showed intracellular spherical viral particles in tubular epithelial cells (Fig. 4). PCR for BKPyV sequenced with ABI PRISM 7900 Sequence Detection System (Applied Biosystems) was positive, with 6 × 10^3^ copies/mL in plasma and 3 × 10^7^ copies/mL in urine. These findings resulted in the diagnosis of BKPyVAN. After diagnosing BKPyVAN, oral prednisolone was tapered. However, kidney function did not improve. On hospital day 87, his IgG level was even lower, at 518 mg/dL after improvement of pneumonia. Intravenous immunoglobulin (IVIg) infusion therapy was performed for BKPyVAN, but the patient’s renal function did not recover. Therefore, he was discharged with maintenance hemodialysis.

## Discussion

BKPyV was first reported in the urine of kidney transplant recipients by Gardner et al. in 1971 [9]. Largely unnoticed during the following decades, BKPyVAN reappeared in the kidney transplantation literature in the late 1990s as a potential cause of graft injury [10]. In early reports, the diagnosis of BKPyVAN in kidney recipients carried a poor prognosis, with 30–58% of such patients experiencing graft dysfunction and premature graft loss [3]. Universal screening and preemptive reduction of immunosuppression are now resulting in less drastic outcomes [11].

BKPyVAN is rare in subjects other than kidney transplant recipients, but an increasing number of cases of BKPyVAN outside of kidney transplant settings are being reported. Shah et al. reported 65 native BKPyVAN cases in diverse populations including solid organ transplant recipients, hematologic transplant recipients, patients with hematologic malignancy, and patients with HIV. The prognosis is poor; of these 65 cases, 14 (21.5%) became chronic dialysis cases and 26 (40.0%) died during follow-up [12]. To our knowledge, there has been no case report of native kidney BKPyVAN in an HTLV-1 carrier.

The mechanism by which ATL patients become immunodeficient involves tumorization of helper T cells associated with HTLV-1 virus infection, suppression by ATL cells of immunoglobulin production by normal B lymphocytes [13], and loss of cytotoxic T cells [14]. Several cases of opportunistic infections have been reported even in the carrier phase of human HTLV-1. The precise mechanisms underlying the immunocompromised status in healthy HTLV-1-infected individuals remain unknown. Funai et al. reported that the response to pokeweed mitogen, which can assess T cell and B cell function, was depressed in HTLV-1 carriers compared to seronegative controls [15]. The case in our report showed low IgG levels. Tashiro et al. reported a low-IgG case of a healthy HTLV-1 carrier with pulmonary cryptococcosis [8]. Takatani et al. reported that HTLV-1 inhibits B cell function by inhibiting B cell activating factor and C-X-C motif ligand 13 concentrations [16]. Hence, low IgG levels in HTLV-1 carriers may reflect the virus’s inhibition of B cell function. In the present case, low IgG levels persisted even after improvement of pneumonia.

Our case was an HTLV-1 carrier with a high anti HTLV-1 antibody titer and a positive PCR for HTLV-1 provirus; however, he did not develop ATL because of a lack of atypical lymphocytes in the peripheral blood. It has been reported that opportunistic infection in HTLV-1 carriers is predictive of the development of ATL [14]. Therefore, careful observation for onset of ATL will be required for this patient.

Effective anti-viral therapy for BKPyVAN is not currently available, and careful reduction of immunosuppression can be effective for treating BKPyVAN in kidney transplant patients [1]. If immunosuppression reduction is not possible, alternative therapies can be considered. Leflunomide, fluoroquinolone, cidofovir, and IVIg treatment have been reported in a small case series for the treatment of BKPyVAN, but none showed efficacy [17]. In the present case of BKPyVAN, IVIg was administered to address low serum IgG levels; however, the patient’s kidney function did not improve.

## Conclusion

HTLV-1 carriers can develop immunodeficiency that allows the development of BKPyVAN in native kidneys even before the onset of ATL. BKPyVAN should be considered when acute kidney injury occurs in a situation complicated by an opportunistic infection.

## Data Availability

KT examined the patient. All authors have full access to all data in this case report. All data generated or analysed during this study are included in this published article [and its supplementary information files].

## Abbreviations

BKPyVAN: BK polyomavirus-associated nephropathy
HTLV-1: Human T-lymphotropic virus type 1
ATL: Adult T cell leukemia
SV: Simian virus
BUN: Blood urea nitrogen
Cr: Creatinine
CRP: C-reactive protein
